# Supplementary material for: CDS-PD: a novel clinical decision support platform for Parkinson’s disease
Source: Sci Rep. 2026 Jan 28;16:6553. doi: 10.1038/s41598-026-37316-1 (PMC12909838; doi:10.1038/s41598-026-37316-1)
Supplement: Supplementary file 1 — Supplementary Material 1 [file 41598_2026_37316_MOESM1_ESM.docx]

Supplementary Appendix

Supplementary Material 1 Title: Full MDS-PD Pseudocode


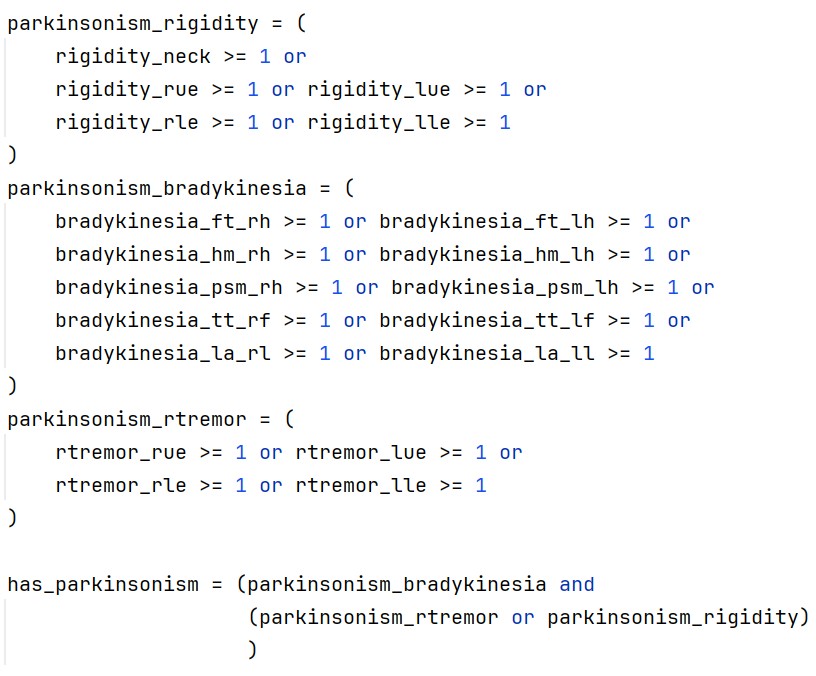


Supplementary Material 1a Caption: Essential Criteria pseudocode


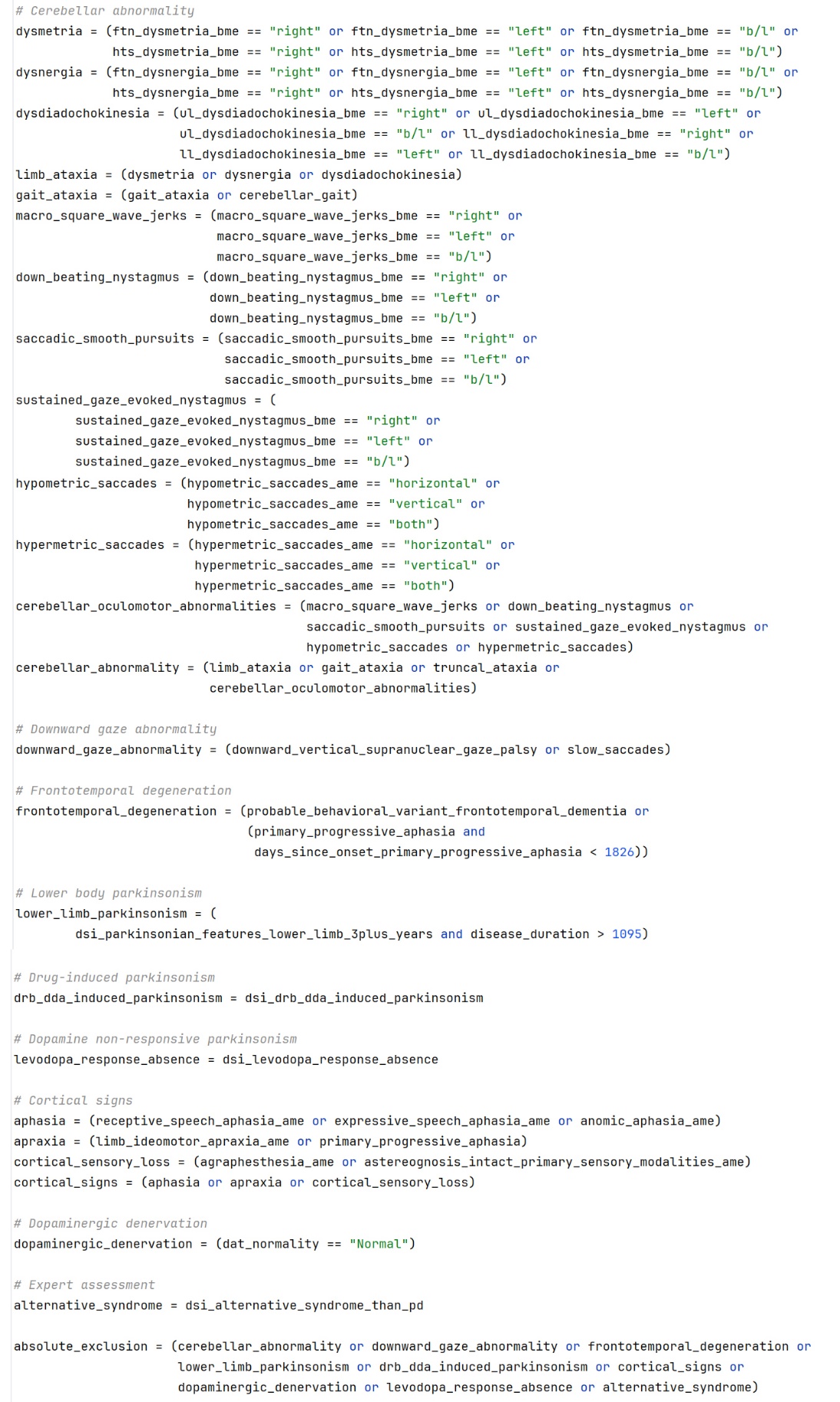


Supplementary Material 1b Caption: Absolute Exclusion Criteria pseudocode


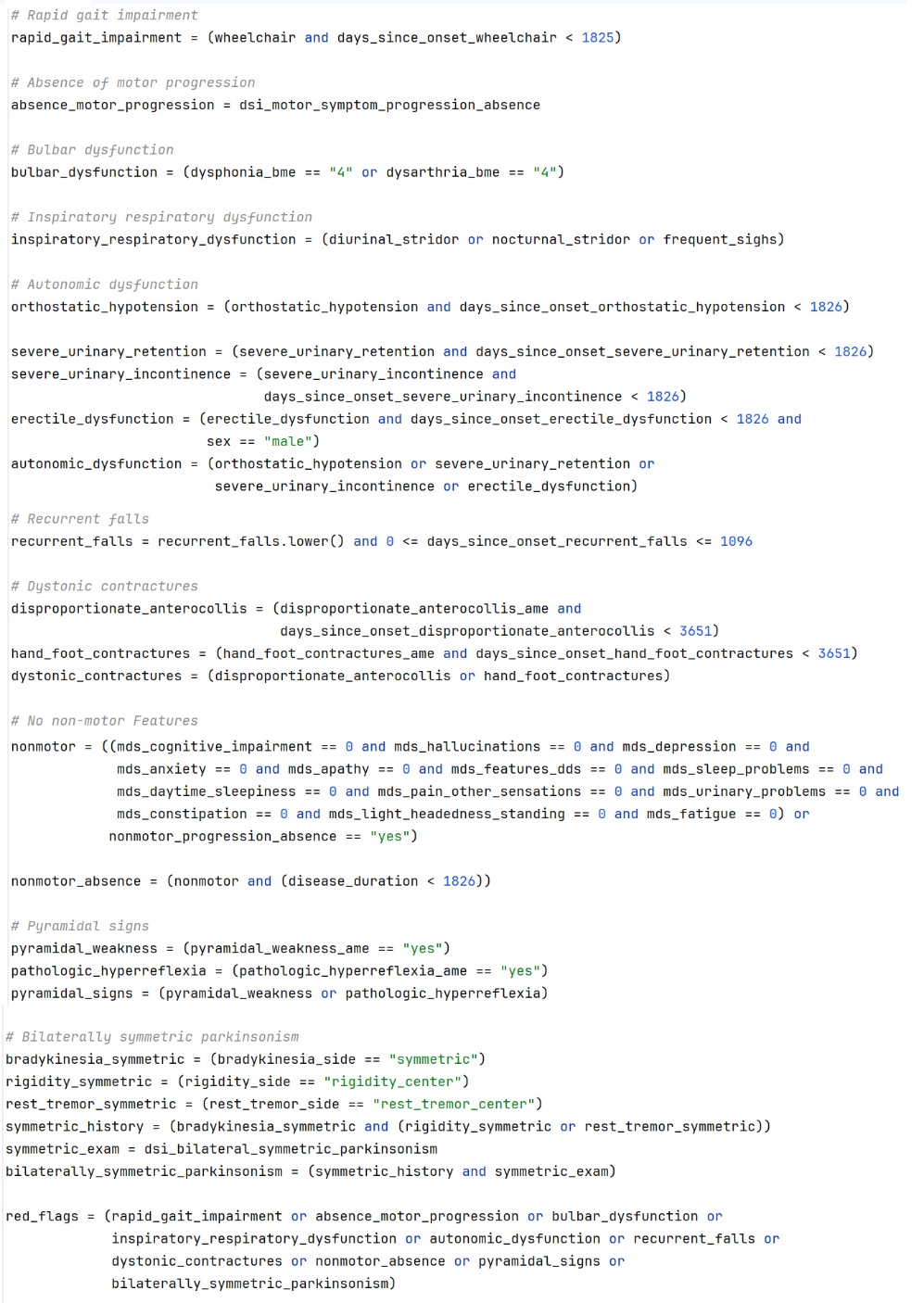


Supplementary Material 1c Caption: Red Flags pseudocode


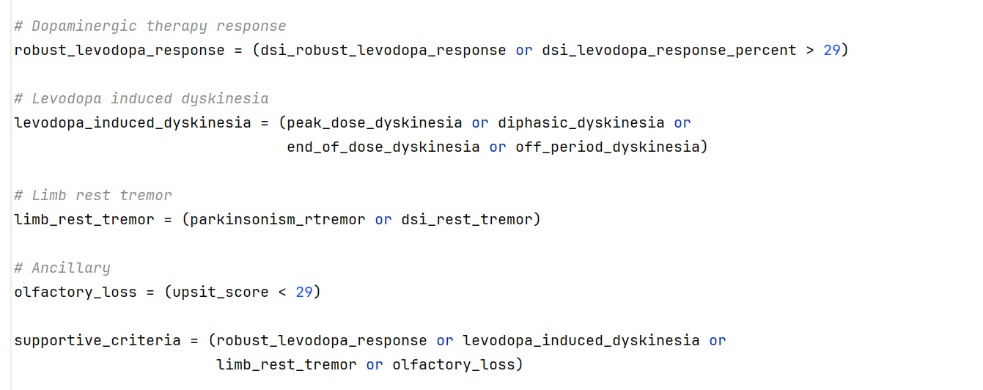


Supplementary Material 1d Caption: Supportive Criteria pseudocode

Supplementary Material 2 Title: CDS-PD Video Walkthrough

(uploaded separately)

Supplementary Material 2 Caption: Example of Patient Information Entry Module and Patient Information Analysis Module. Note: Test0507 shown is a dummy/test subject.

Supplementary Material 3 Title: CDS-PD Early PD Module


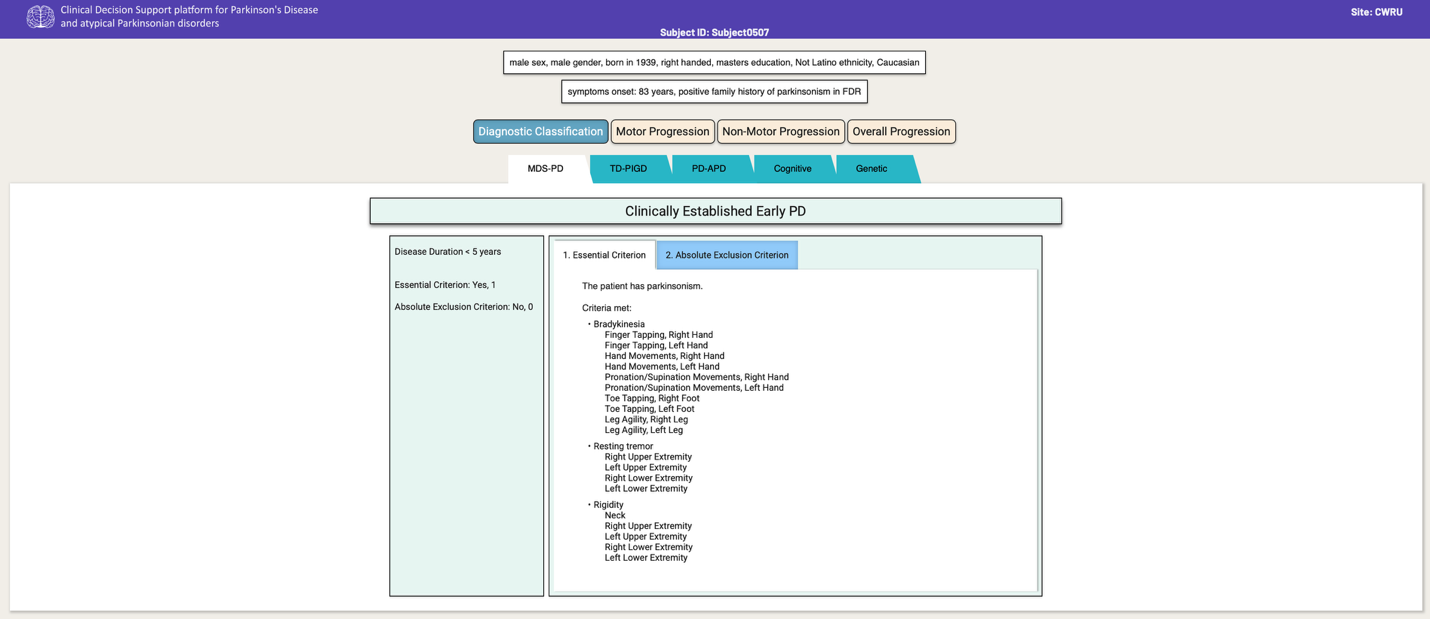


Supplementary Material 3 Caption: Functional analysis module applying the Movement Disorder Society Criteria for Clinically Established Early Parkinson’s Disease for patients with disease duration of < 5 years. Note: Subject0507 shown is a dummy/test subject.

Supplementary Material 4 Title: CDS-PD Clinical Subtype Module


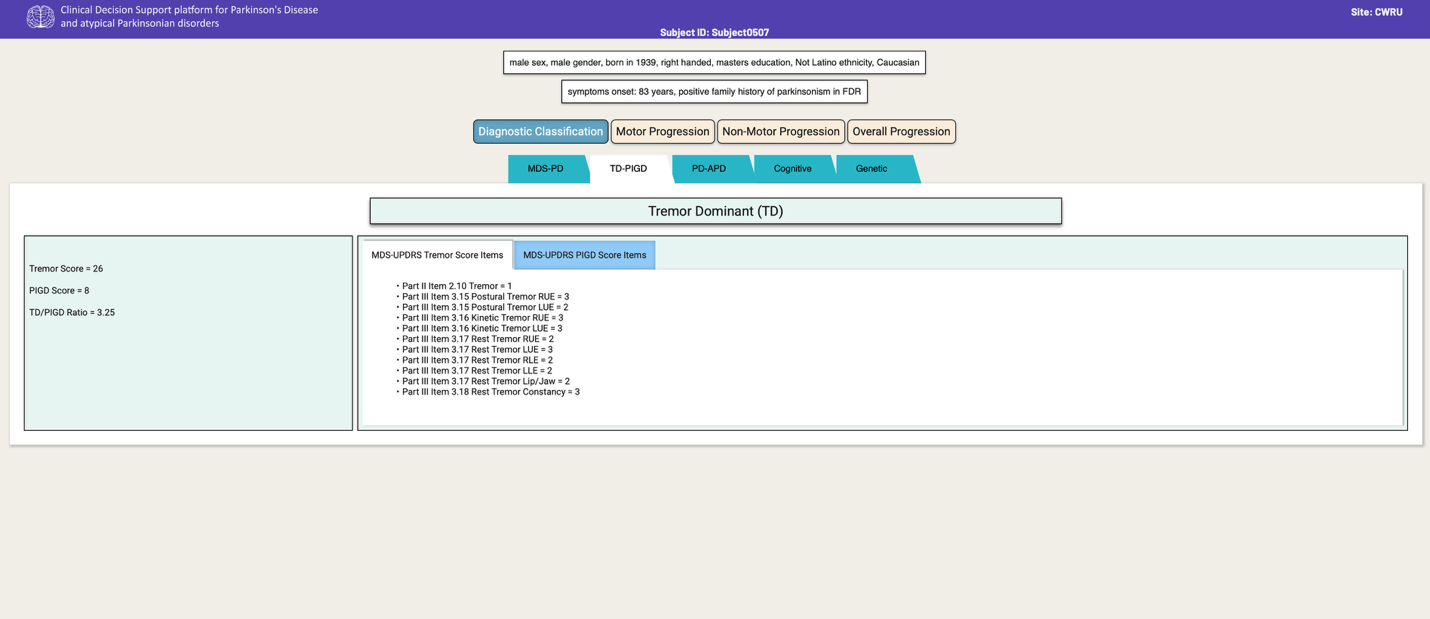


Supplementary Material 4 Caption: Functional analysis module applying the Tremor Dominant / Postural Instability Gait Dysfunction / Indeterminate subtyping based on MDS-UPDRS. Note: Subject0507 shown is a dummy/test subject.
